# Association of smoking, alcohol, and coffee consumption with the risk of ovarian cancer and prognosis: a mendelian randomization study

**DOI:** 10.1186/s12885-023-10737-1

**Published:** 2023-03-20

**Authors:** Sicong Liu, Songwei Feng, Furong Du, Ke Zhang, Yang Shen

**Affiliations:** 1 Department of Obstetrics and Gynecology, Zhongda Hospital, School of Medicine, Southeast University, Nanjing, 210009 China; 2 State Key Laboratory of Translational Medicine and Innovative Drug Development, Jiangsu Simcere Diagnostics Co., Ltd, Nanjing, Jiangsu 210042 China; 3 Department of Obstetrics and Gynecology, Zhongda Hospital, School of Medicine, Southeast University, Nanjing, 210009 China

**Keywords:** Smoking, Alcohol, Coffee, Ovarian cancer, Mendelian randomization, Prognosis

## Abstract

**Objective:**

Currently, the association between smoking, alcohol, and coffee intake and the risk of ovarian cancer (OC) remains conflicting. In this study, we used a two-sample mendelian randomization (MR) method to evaluate the association of smoking, drinking and coffee consumption with the risk of OC and prognosis.

**Methods:**

Five risk factors related to lifestyles (cigarettes per day, smoking initiation, smoking cessation, alcohol consumption and coffee consumption) were chosen from the Genome-Wide Association Study, and 28, 105, 10, 36 and 36 single-nucleotide polymorphisms (SNPs) were obtained as instrumental variables (IVs). Outcome variables were achieved from the Ovarian Cancer Association Consortium. Inverse-variance-weighted method was mainly used to compute odds ratios (OR) and 95% confidence intervals (Cl).

**Results:**

The two-sample MR analysis supported the causal association of genetically predicted smoking initiation (OR: 1.15 per SD, 95%CI: 1.02–1.29, *P* = 0.027) and coffee consumption (OR: 1.40 per 50% increase, 95%CI: 1.02–1.93, *P* = 0.040) with the risk of OC, but not cigarettes per day, smoking cessation, and alcohol consumption. Subgroup analysis based on histological subtypes revealed a positive genetical predictive association between coffee consumption and endometrioid OC (OR: 3.01, 95%CI: 1.50–6.04, *P* = 0.002). Several smoking initiation-related SNPs (rs7585579, rs7929518, rs2378662, rs10001365, rs11078713, rs7929518, and rs62098013), and coffee consumption-related SNPs (rs4410790, and rs1057868) were all associated with overall survival and cancer-specific survival in OC.

**Conclusion:**

Our findings provide the evidence for a favorable causal association of genetically predicted smoking initiation and coffee consumption with OC risk, and coffee consumption is linked to a greater risk of endometrioid OC.

**Supplementary Information:**

The online version contains supplementary material available at 10.1186/s12885-023-10737-1.

## Introduction

Ovarian cancer (OC) is the seventh most common malignancy in women worldwide, with the highest mortality rate among gynecological tumors [1]. According to statistics, the 5-year survival rate of patients with OC ranges from 30 to 50% [2]. In 2022, approximately 19,880 people will be diagnosed with OC, and roughly 12,810 will die in the United States [3]. Despite awareness of OC, curative and survival trends have not dramatically changed because there still exists a challenge for early diagnosis.

Until now, the extract pathogenesis of OC development remains unknown, which may be mediated by a variety of factors including genetics, environment, and lifestyles. A previous study has shown an unexpected phenomenon that several lifestyle behaviors, such as smoking, drinking alcohol, and coffee consumption, are significantly associated with increased OC risk [4]. Meanwhile, smoking and alcohol consumption have positive associations with a poor prognosis of OC, probably because these behaviors themselves have a detrimental effect on survival [5, 6]. However, some studies have discovered a neutral or poor relationship between smoking, alcohol, and OC [7–9]. Similarly, the relationship between coffee intake and OC is also contradictory [10, 11]. Due to the possible residual confounders and lack of high-quality randomized controlled trials, whether there is a causal relationship between smoking, alcohol, coffee intake and OC needs to be investigated urgently.

Mendelian randomization (MR) is a method for determining whether a certain exposure has a causal effect on an outcome [12]. To reduce confounders and reversed causation in observational data, MR design utilizes single-nucleotide polymorphisms (SNPs) as instrumental variants (IVs) for risk factors [13]. Since MR relies on random assignment of alleles during meiosis, it is less affected by confounding factors and can reverse causality. Summary-level data from genome-wide association studies (GWAS) are easier to be obtained and typically large for two-sample MR design, which can enhance the genetic interpretation of IVs on exposure and improve the accuracy and reliability of analysis results [14].

In the present study, we evaluated the association between smoking, drinking and coffee consumption and the risk of OC using a two-sample MR method, aiming at determining whether these lifestyles have a causal rather than pleiotropic impact on OC. Additionally, we also assessed the association of these genetically predicted exposures with the OC prognosis.

## Materials and Methods

### Study design

**Fig. 1:**
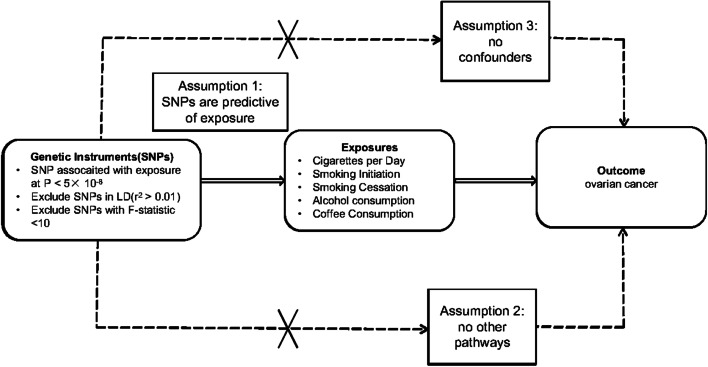
Genetic instrument construction, data sources, and analysis plan on the association between lifestyle factors and ovarian cancer. SNP: single nucleotide polymorphisms

In this MR study, the SNPs were retrieved from a number of published GWAS to determine the causal relationship between exposures and outcomes. Three important assumptions needed to be proven in order to guarantee an efficient MR analysis process: (1) The SNPs were linked with smoking, drinking, and coffee consumption; (2) The SNPs only impacted OC via smoking, drinking, and coffee consumption; (3) The SNPs were entirely unconnected with any possible confounding variables that affected smoking, drinking, and coffee consumption as well as OC. The assumptions of the IVs are shown in Fig. 1.

### Genetic Instrument Selection and Data Sources

Independent SNPs were chosen from a large meta-analysis including 33 GWAS and a genome-wide meta-analysis involving 28 studies, respectively [15, 16]. They were associated with the number of cigarettes per day (*n* = 337,334), smoking initiation (whether an individual had ever smoked regularly, *n* = 1,232,091), smoking cessation (current versus former smokers, *n* = 547,219), drinks per week (*n* = 66,450), and coffee consumption (*n* = 375,833). Instruments were chosen at the genome-wide significance threshold (*P* < 5 × 10^–8^) for each exposure trait. The minor allele frequency (MAF) threshold was set at 0.3. We clumped linkage disequilibrium based on European ancestry reference data (1000 Genomes Project, *r*^2^ = 0.01, clump window = 10,000 kb) to establish the independence among the SNPs used. Palindromic SNPs were removed from the instrumental variables.

We chose independent SNPs for each exposure trait that were significant at the genome-wide level in each GWAS (*P* = 5 × 10^–8^). For each selected SNP, we identified the pleiotropy by Phenoscanner (http://www.phenoscanner.medschl.cam.ac.uk/). Pleiotropic SNPs were included in the analysis, and subsequently excluded if sensitivity analysis revealed horizontal pleiotropy. To avoid bias caused by weak instrumental variables, we calculated the *F* statistic of each SNP. Generally, *F* > 10 often indicates there is no weak instrument bias. Finally, 189 independent SNPs were chosen as the IVs for MR analysis, including 25 SNPs for cigarettes per day, 88 SNPs for smoking initiation, 7 SNPs for smoking cessation, 37 SNPs for alcohol drinking and 32 SNPs for coffee consumption. The specifics of instrument selection and the corresponding genome-wide association meta-analyses are shown in Table 1 and the raw information of the associations of selected SNPs with lifestyle behaviors are given in Supplementary Table S1.

**Table 1:** Data sources of instrumental variables

Traits	No. of participants	Ancestry	Unit for each factor	No. of variants included	Number of SNPs available^a^	Number of SNPs used^b^	PubMed ID
Cigarettes per day	337,334	European	SD increase in the number of cigarettes per day	11,991,601	2,129	25	30,643,251
Smoking initiation	1,232,091	European	Previous smoking compared with never smoking	11,792,288	7,846	88	30,643,251
Smoking cessation	547,219	European	Current smokers Compared with former smokers	12,186,231	223	7	30,643,251
Alcohol consumption	66,450	European	SD increase in log-transformed alcoholic drinks/week	11,976,706	5,196	37	30,643,251
Coffee consumption	375,833	European	50% change	7,875,318	2,996	32	31,046,077
Ovarian cancer	66, 450	European	-	-	-	-	28,346,442

“a” corresponds to the number of SNPs available at the genome-wide significance level (P < 5 × 10^–8^). “b” corresponds to the number of SNPs (or linkage disequilibrium proxies) available in ovarian cancer datasets

*SNP* Single nucleotide polymorphism, *SD* Standard deviation

We used GWAS summary data for the overall OCs and subtypes from Ovarian Cancer Association Consortium (OCAC) [17], an international collaboration with participants of European ancestry recruited from 14 countries, to determine whether genetically predicted smoking, drinking, and coffee consumption was associated with the risk of OC. The studies included 66,450 samples from 7 different genotyping projects, while the OCAC OncoArray data contained 63 genotyping project/case–control combinations. Individuals in the OCAC were disqualified if 5% or more of the genotyping calls were missing. The summary data (25,509 cases; 40,941 controls) were used to analyze the connection of these SNPs with the risk of total OCs. 13,037 high-grade serous, 1,012 low-grade serous, 1,366 clear cell, 2,810 endometrioid, and 1,417 invasive mucinous OC samples were available for the subgroup analyses. Our study only utilized the results of published GWAS. All summary data were downloaded from the IEU OpenGWAS project (https://gwas.mrcieu.ac.uk/).

### Statistical analysis

To identify the genetic relationships from each separate GWAS dataset, we employed a two-sample MR design. Inverse variance weighted (IVW) with random effects served as the primary method of statistical analysis. The advantage of the random effects model is that it accounts for the variations in the effect sizes of the chosen SNPs on the exposed phenotypes [18]. The IVW approach, which is used on the presumption that all SNPs are valid IVs and independent of each other [13], is for meta-summarizing the effects of various loci in MR analysis of multiple SNPs. To prevent the influence of unidentified and immeasurable confounders, MR regression, weighted median, weighted mode, and simple mode were utilized as supplemental analyses. The MR-Egger regression can identify and correct the potential pleiotropy [19], but may reduce statistical power. The weighted median method provides a consistent estimate of causality, even though more than half of the instrument weights originate from invalid IVs [20]. Weighted model method weights the contribution of each variant to the clustering by the inverse variance associated with its results [21]. The simple model method groups the SNPs depending on the similarity of MR associations. MR estimates are unbiased when the maximally clustered SNPs are valid instruments [22]. Using MR-Egger regression and weighted medians, sensitivity analysis was performed to clarify possible breaches of the assumptions related to the instrumental variable. The *P* value for the intercept in MR-Egger regression was employed as an index of pleiotropy [20]. MR-PRESSO detects the presence of horizontal pleiotropy, removes possible outliers and estimates the corrected results, testing for differences between pre-corrected and post-corrected results [23]. Cochran Q values were used to assess the heterogeneity among selected IVs for each exposure.

In order to determine whether a particular genetic variation was responsible for the causal connection, the "leave-one-out" method for sensitivity analysis was used. Based on 5 histological subtypes of OCAC, we evaluated the association between the risk factors and the risk of OC subtypes. Additionally, we also evaluated the causative impact of selected SNPs on the prognosis of OC, in accordance with a MR analytic framework basing on SUrvival related cancer Multi-omics database via MEndelian Randomization (SUMMER, http://njmu-edu.cn:3838/SUMMER/) [24]. All statistical analyses were carried out using the package “TwoSampleMR" and “MR-PROESSO” in R software 4.2.0, and all *P* values were two-sided.

## Results

### Association of smoking, alcohol and coffee consumption with the overall risk of OC

**Fig. 2:**
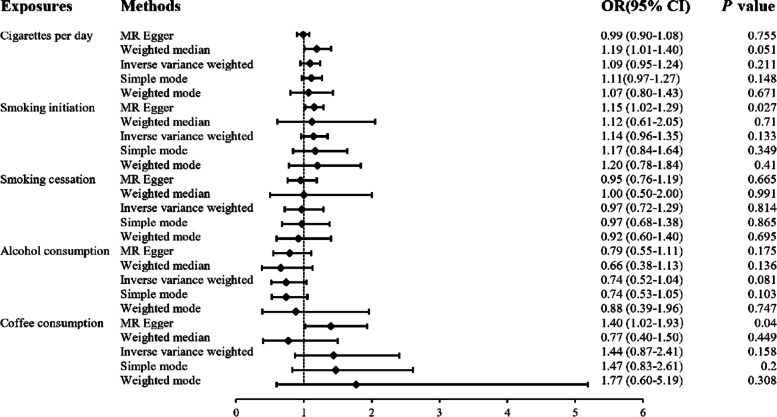
Association of smoking, alcohol and coffee consumption with the overall risk of ovarian cancer in the Mendelian randomization analysis. OR, odds ratio; CI, confidence interval

The *F* statistics for the five risk factors analyzed in this MR study varied from 41 to 642, indicating that there may not have been any weak instrument biases in our analyses. There were no associations of genetic liability to cigarettes per day, smoking cessation and alcohol consumption with the overall risk of OC. However, two-sample MR showed an adverse effect of smoking initiation and coffee consumption on the overall risk of OC (OR: 1.15, 95%CI: 1.02–1.29, *P* = 0.027; OR: 1.40, 95%CI: 1.02–1.93, *P* = 0.040; Fig. 2, Table 2).

**Table 2:** Mendelian randomization estimates between phenotypes and ovarian cancer

Exposures	Outcomes	Method	OR (95%CI)	*P* value
Cigarettes per day	Overall ovarian cancer	Inverse-variance weighted	0.99 (0.90–1.08)	0.755
		MR Egger	1.19 (1.01–1.40)	0.051
		Weighted median	1.09 (0.95–1.24)	0.211
		Weighted mode	1.11(0.97–1.27)	0.148
		Simple mode	1.07 (0.80–1.43)	0.671
Smoking initiation	Overall ovarian cancer	Inverse-variance weighted	1.15 (1.02–1.29)	0.027
		MR Egger	1.12 (0.61–2.05)	0.71
		Weighted median	1.14 (0.96–1.35)	0.133
		Weighted mode	1.17 (0.84–1.64)	0.349
		Simple mode	1.20 (0.78–1.84)	0.41
Smoking cessation	Overall ovarian cancer	Inverse-variance weighted	0.95 (0.76–1.19)	0.665
		MR Egger	1.00 (0.50–2.00)	0.991
		Weighted median	0.97 (0.72–1.29)	0.814
		Weighted mode	0.97 (0.68–1.38)	0.865
		Simple mode	0.92 (0.60–1.40)	0.695
Alcohol consumption	Overall ovarian cancer	Inverse-variance weighted	0.79 (0.55–1.11)	0.175
		MR Egger	0.66 (0.38–1.13)	0.136
		Weighted median	0.74 (0.52–1.04)	0.081
		Weighted mode	0.74 (0.53–1.05)	0.103
		Simple mode	0.88 (0.39–1.96)	0.747
Coffee consumption	Overall ovarian cancer	Inverse-variance weighted	1.40 (1.02–1.93)	0.04
		MR Egger	0.77 (0.40–1.50)	0.449
		Weighted median	1.44 (0.87–2.41)	0.158
		Weighted mode	1.47 (0.83–2.61)	0.2
		Simple mode	1.77 (0.60–5.19)	0.308

The complementary estimates further demonstrated this consistency in detecting smoking initiation (Table 3). MR-PRESSO did not show a horizontal pleiotropy. There may be no pleiotropy since the *P* value for the MR-Egger intercept was over 0.05 (Table 3). No heterogeneity was detected and the leave-one-out sensitivity test indicated that the results of the MR analysis were robust.

**Table 3:** Pleiotropy and heterogeneity of five phenotypes in ovarian cancer

Exposures	Pleiotropy (*P* value)	Heterogeneity (*P* value)
Cigarettes per day	0.012	0.505
Smoking initiation	0.946	0.277
Smoking cessation	0.875	0.793
Alcohol consumption	0.397	1.26E-05
Coffee consumption	0.053	0.809

### Association of smoking initiation and coffee consumption with OC subtypes

Based on histological subtypes, a subgroup analysis of OC was performed in terms of smoking initiation and coffee consumption. The results showed that coffee consumption was strongly associated with a high risk of endometrioid OC (OR: 3.01, 95%CI: 1.50–6.04, *P* = 0.002), but not other OC subtypes (Table 4). Additionally, no association was observed between genetically predicted smoke initiation and all OC subtypes.

**Table 4:** Causal estimates for the association between smoking initiation and coffee consumption and ovarian cancer subtypes

Exposures	Histological subtypes	OR (95%CI)	*P* value
Smoking initiation	High-grade serous	1.15 (1.00–1.33)	0.057
Smoking initiation	Low grade serous	1.43 (0.93–2.20)	0.104
Smoking initiation	Clear cell	1.24 (0.87–1.76)	0.231
Smoking initiation	Endometrioid	1.13 (0.86–1.48)	0.369
Smoking initiation	Invasive mucinous	0.92 (0.65–1.32)	0.667
Coffee consumption	High-grade serous	1.23 (0.84–1.81)	0.28
Coffee consumption	Low grade serous	1.00 (0.28–3.53)	0.998
Coffee consumption	Clear cell	2.02 (0.73–5.61)	0.178
Coffee consumption	Endometrioid	3.01 (1.50–6.04)	0.002
Coffee consumption	Invasive mucinous	1.15 (0.44–3.05)	0.77

### Effect of smoking initiation and coffee consumption on the prognosis of OC

**Fig. 3:**
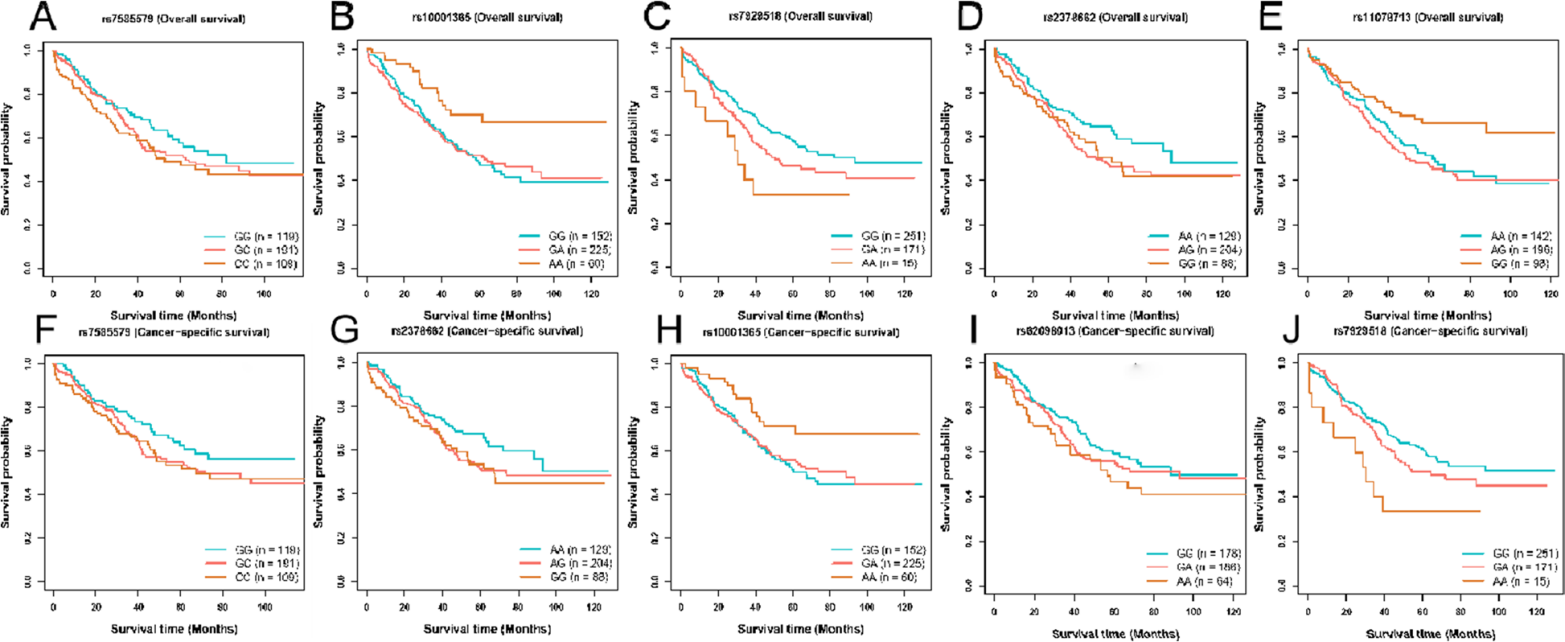
Kaplan–Meier plots of the effect of smoking initiation on overall survival and cancer-specific survival in ovarian cancer. Association between **A** rs7585579, **B** rs10001365, **C** rs7929518, **D** rs2378662, **E** rs11078713 and overall survival in ovarian cancer. Association between **F** rs7585579, **G** rs2378662, **H** rs10001365, **I** rs62098013, **J** rs7929518 and cancer-specific survival in ovarian cancer

**Fig. 4:**
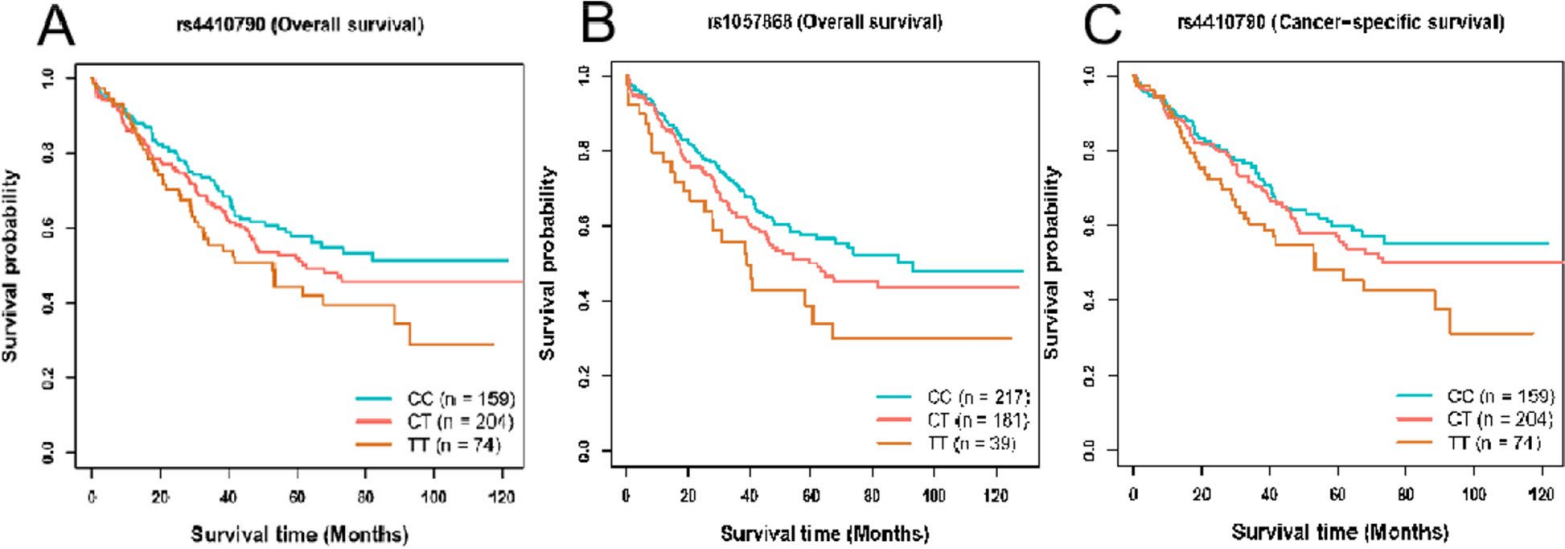
Kaplan–Meier plots of the effect of coffee consumption on overall survival and cancer-specific survival in ovarian cancer. Association between **A** rs4410790, **B** rs1057868 and overall survival in ovarian cancer. Association between **C** rs4410790 and cancer-specific survival in ovarian cancer

We evaluated the effect of the chosen SNPs on the prognosis of OC, which were discovered to be linked with a greater probability of developing OC using MR analysis. Shorter overall survival (OS) for OC was positively correlated with SNPs rs7585579 (HR: 1.25, *P* = 0.031), rs7929518 (HR: 1.45, *P* = 0.004), and rs2378662 (HR: 1.26, *P* = 0.030) related to smoking initiation, whereas SNPs rs10001365 (HR: 0.74, *P* = 0.007) and rs11078713 (HR: 0.80, *P* = 0.033) related to smoking initiation had the opposite effect. Additionally, SNPs rs7585579 (HR: 1.30, *P* = 0.018), rs7929518 (HR: 1.42, *P* = 0.013), rs2378662 (HR: 1.26, *P* = 0.037) and rs62098013 (HR: 1.24, *P* = 0.045) associated with smoking initiation were also identified to be linked with shorter cancer-specific survival (CSS), whereas rs10001365 (HR: 0.75, *P* = 0.016) was significantly associated with longer CSS in OC. Notably, SNPs rs4410790 (HR: 1.31, *P* = 0.007) and rs1057868 (HR: 1.28, *P* = 0.022) related to coffee consumption were both correlated with poor OS in OC, and SNP rs4410790 (HR: 1.30, *P* = 0.016) was in the association with poor CSS in OC (Table 5; Figs. 3 and 4).

**Table 5:** Effect of smoking initiation and coffee consumption on overall survival and cancer-specific survival in all ovarian cancers

SNPs	Exposures	HR_OS	SE-OS	*P*_value_OS	HR_CSS	SE-CSS	*P*_value_CSS
rs7585579	Smoking initiation	1.25	0.1	0.031	1.3	0.11	0.018
rs10001365	Smoking initiation	0.74	0.11	0.007	0.75	0.12	0.016
rs7929518	Smoking initiation	1.45	0.13	0.004	1.42	0.14	0.013
rs2378662	Smoking initiation	1.26	0.1	0.03	1.26	0.11	0.037
rs11078713	Smoking initiation	0.8	0.1	0.033	0.85	0.11	0.126
rs62098013	Smoking initiation	1.2	0.1	0.08	1.24	0.11	0.045
rs4410790	Coffee consumption	1.31	0.1	0.007	1.3	0.11	0.016
rs1057868	Coffee consumption	1.28	0.11	0.022	1.14	0.12	0.261

*Abbreviations*: *SNP* Single nucleotide polymorphism, *HR* Hazard ratio, *OS* Overall survival, *CSS* Cancer-specific survival

## Discussion

The prevention of OC remains a big challenge at present. The results of this two-sample MR analysis suggested that smoking initiation and coffee consumption were associated with an increased risk of OC. The MR analysis failed to provide the evidence for a causal effect of alcohol drinking, cigarettes per day and smoking cessation. The subgroup analysis based on OC subtypes further revealed that coffee consumption was associated with a higher risk of developing endometrioid OC. Notably, several SNPs related to smoking initiation and coffee consumption were found to relate to the OS and CSS of OC.

Smoking is a predictor of cancer incidence and related to worse long-term outcomes [25, 26]. Our results offered a modest evidence for a positive causal relationship between smoking initiation and OC risk according to the IVW analysis, and there was no relationship between cigarettes per day or smoking cessation and OC risk. According to a recent research involving 1,279 participants, the risk of OC-specific mortality has risen by 19% and 21% in patients with pre-diagnosis and post-diagnosis smoking compared with never smokers, respectively [27]. By comparison to women who had never smoked, the overall prevalence of OC was only marginally higher among current smokers. Smoking initiation may be a causative risk for the total OC, which was supported by this MR study based on the summary data from 66,450 women. There is also evidence suggesting that women who had never smoked previously were more likely to develop mucinous OC than those who did [9]. The current research, however, could not corroborate this finding linking smoking with invasive mucinous OC.

The precise mechanisms of how smoking contributes to the development of OC are not fully understood. There are various carcinogens in cigarette smoke, including N-nitrosamines, aromatic amines, 1,3-butadiene, and benzene [28, 29]. Nicotine in tobacco is a cancer promoter, and chronic smoking may promote cancer cell proliferation, epithelial-mesenchymal transition and angiogenesis [30–33], as well as cause OC to develop a more aggressive phenotype for promoting metastatic spread [34]. Smoking may enhance the pro-inflammatory cytokines and chemokines in the tumor environment, consequently increasing the likelihood of treatment resistance [35]. Smokers are more prone to engage in unhealthy lifestyles including obesity and alcohol use, which may have a negative impact on the prognosis of OC, even though alcohol consumption is not linked with OC in this MR analysis. Currently, smoking cessation has been described as a key method in preventing a variety of cancers [36].

Our finding shows that there is no relationship between alcohol consumption and OC, which is consistent with previously published studies. A meta-analysis that included 16,554 ovarian cancer patients found that alcohol consumption was not associated with OC risk [37]. In addition, Cook et al. [38] suggested that alcohol consumption consistent with guidelines did not increase the risk of epithelial OC, but higher wine consumption was associated with a lower risk of ovarian cancer. The biological mechanisms underlying the relationship between alcohol and OC are currently unclear. Alcohol consumption may lead to increased cumulative estrogen exposure, leading to the development of oc through epithelial cell genotoxicity and mitosis [39, 40]. Meanwhile, acetaldehyde, an oxidized metabolite of alcohol, can be carcinogenic [41]. On the contrary, polyphenols, flavonoids, and resveratrol found in alcohol such wine and red wine had anti-inflammatory and antioxidant effects, which may decrease OC risk [42–44].

There is a correlation between coffee consumption and the risk of developing OC, although there is no statistically significant relationship, according to a systematic review of 15 OC studies including 5,021 individuals [45]. Previous studies have demonstrated that caffeine intake among premenopausal women is associated with increased OC risk, whereas there is no or little association among postmenopausal women [36, 46]. In our two-sample MR analysis, coffee drinking was positively related to the risk of OC. However, several studies showed that drinking coffee did not increase or lower the incidence of OC [47–49]. Additionally, we also found the association between coffee drinking as a risk factor and the risk of endometrioid OC. Ong et al. discovered no evidence indicative of a causal relationship between genetically predicted coffee or caffeine concentrations and epithelial OC risk [50], which was conflicting with our two-sample MR research. There were several reasons that may explain this phenomenon. First, the database we chose was the latest GWAS database with a larger sample size, thus a large variety of IVs may be chosen, consequently increasing the potential of the association between genetically interpreted coffee consumption and OC risk. In our two-sample MR analysis, a total of 28 significant SNPs were selected for coffee consumption, significantly more than those in the prior study [51]. Second, the Ong’s study only examined the connection between coffee intake and epithelial OC, but in our study, the potential causal relationship between coffee consumption and OC subtypes was investigated. Interestingly, we found a strong association between coffee consumption and endometrioid OC risk. Acrylamide, which is produced during roasting coffee beans at high temperatures, may be the mechanism causing this outcome [52]. High acrylamide intake may be scientifically conceivable as a potential risk factor for OC [53]. In addition, caffeine is able to inhibit aromatase activity and increase the secretion of sex hormone-binding globulin altering the hormonal milieu [54, 55]. The hormonal alterations synergize with coelomic metaplasia, proliferation of progenitor stem cells, or retrograde menstruation of endometrial cells, leading to implantation and proliferation of ectopic endometrial cells and increasing the risk of endometrioid ovarian cancer [56].

It is well known that obesity is a key risk factor for OC. In our study, robust evidences points to favorable causal association of genetically predicted smoking initiation and coffee consumption with OC risk. Caffeine is the main ingredient in coffee [50]. It have been shown that in obese people the apparent distribution of caffeine increases by 60%, but does not affect caffeine clearance [57]. Biologically active compounds in coffee, such as chlorogenic acid, caffeine, have shown to be associated with anti-obesity benefits [58]. Nicotine in tobacco can increase energy consumption and inhibit appetite in a short period of time, but people who smoke more have a higher BMI than light smokers, probably because heavy smokers are accompanied by unhealthy behaviors such as poor diet, alcohol abuse and low physical activity [59, 60]. Also, smoking can promote visceral fat accumulation and insulin resistance and hyperinsulinemia, increasing the risk of obesity [59].

The IVW method's statistical power is much greater than that of other MR methods, particularly MR-Egger [61]. In our study, IVW was used as a main approach to screen the results in MR. To ensure the robust findings, we also performed a sensitivity analysis. Taken together, our findings supported the hypothesis that smoking and coffee consumption could increase the risk of OC, thus the strategies to reduce the exposure of these two factors was worthy of attention to decrease the risk of OC. Regular smoking and coffee cessation campaigns should be conducted among the female population to lower the incidence of OC.

The present study has several strengths and limitations. First, the majority of studies on smoking, drinking, and coffee utilized self-reported consumption, which was easy to cause the bias. This MR study, however, examined summary statistics of several behaviors from a large dataset. In our two-sample MR investigation, genetic variation may examine the possible causative influence of exposures on OC without being biased or confounded by confounding or reserve causation [61]. Second, we explored the association of a few significant SNPs with OS and CSS in OC and made Kaplan–Meier plot diagrams to show. Additionally, all of the studies included communities with a predominance of European ancestry, which reduced the possibility of population stratification-related bias, but may not be generalizable to other groups. Pleiotropy was ruled out, but there might still be alternative mechanisms through which SNPs and OC were related. Importantly, we cannot rule out a genetic link between OC, smoking initiation, and coffee consumption. Notably, the menopausal status (premenopausal or postmenopausal) of OC patients was not stratified in this research, so it was uncertain to determine whether the effect of smoking and coffee intake on the OC risk was under the influence of menopausal status. Another limitation was that a two-sample MR design was unable to evaluate the reverse causality across these exposures on OC.

## Conclusions

This two-sample MR study provided the evidence for favorable causal association between genetically predicted smoking initiation and coffee consumption and OC risk. Meanwhile, coffee consumption was linked to a greater risk of endometrioid OC according to histological subgroup analysis. In the future, clinicians can collect peripheral blood or tumor tissue from OC patients for SNP testing. They can use several methods for SNP detection, such as sequencing, TaqMan probes, gene microarrays, and mass spectrometry. Based on our results, the risk of OC and survival outcomes can be better identified.

## Supplementary Information

**Additional file 1: Table S1.** The causal effect estimates of the associations between genetic instrumental variables for lifestyle behaviors and risk of ovarian cancers.

## Data Availability

The datasets analyzed in this study are publicly available summary statistics. GWAS summary statistics for smoking, alcohol and coffee consumption are available at https://conservancy.umn.edu/handle/11299/201564 and https://digitalhub.northwestern.edu/catalog?depositor=%22mcc340%22&search_field=advanced. The data of overall ovarian cancer(ID: ieu-a-1120), high grade serous ovarian cancer(ID: ieu-a-1121), low grade serous ovarian cancer(ID: ieu-a-1122), clear cell ovarian cancer(ID: ieu-a-1124), endometrioid ovarian cancer(ID: ieu-a-1125) and invasive mucinous ovarian cancer(ID: ieu-a-1123) can be obtained from https://gwas.mrcieu.ac.uk/.

## Abbreviations

CSS: Cancer-specific survival
HR: Hazard ratio
IVs: Instrumental variables
IVW: Inverse variance weighted
LD: Linkage disequilibrium
MR: Mendelian randomization
OC: Ovarian Cancer
OCAC: Ovarian Cancer Association Consortium
OR: Odds ratio
OS: Overall survival
SNP: Single nucleotide polymorphism
