# Supplementary material for: Association of smoking, alcohol, and coffee consumption with the risk of ovarian cancer and prognosis: a mendelian randomization study
Source: BMC Cancer. 2023 Mar 20;23:256. doi: 10.1186/s12885-023-10737-1 (PMC10026459; doi:10.1186/s12885-023-10737-1)
Supplement: Supplementary file 1 — Additional file 1: Table S1. The causal effect estimates of the associations between genetic instrumental variables for lifestyle behaviors and risk of ovarian cancers. [file 12885_2023_10737_MOESM1_ESM.doc]

**Table S1 The causal effect estimates of the associations between genetic instrumental variables for lifestyle behaviors and risk of ovarian cancers**

| Trait | SNP | Gene | Effect_allele | F_statistic | Exoposure | | Outcome:ovarian cancer | |
| --- | --- | --- | --- | --- | --- | --- | --- | --- |
|  |  |  |  |  | beta | se | beta | se |
| Cigarettes Per Day | rs111240159 | CTC-490E21.12 | T | 41.395 | 0.139 | 0.024 | 0.012 | 0.067 |
| Cigarettes Per Day | rs11671669 | CTC-490E21.12 | A | 65.467 | -0.149 | 0.015 | -0.005 | 0.043 |
| Cigarettes Per Day | rs11725618 | AC112518.3 | C | 162.394 | 0.036 | 0.006 | -0.011 | 0.015 |
| Cigarettes Per Day | rs13141210 | AC112518.3 | T | 181.336 | -0.031 | 0.006 | -0.008 | 0.014 |
| Cigarettes Per Day | rs2084533 | PLCL2 | T | 169.463 | 0.034 | 0.006 | 0.000 | 0.014 |
| Cigarettes Per Day | rs215600 | PDE1C | A | 173.836 | -0.049 | 0.006 | 0.010 | 0.014 |
| Cigarettes Per Day | rs2273500 | CHRNA4 | C | 128.278 | 0.068 | 0.008 | 0.006 | 0.019 |
| Cigarettes Per Day | rs2386571 | C16orf97 | C | 179.709 | -0.032 | 0.006 | -0.002 | 0.013 |
| Cigarettes Per Day | rs3025383 | DBH | C | 141.940 | -0.058 | 0.007 | 0.019 | 0.018 |
| Cigarettes Per Day | rs4785587 | SPATA2L | A | 180.669 | -0.034 | 0.006 | 0.013 | 0.013 |
| Cigarettes Per Day | rs56113850 | CYP2A6 | C | 178.453 | 0.107 | 0.006 | 0.004 | 0.015 |
| Cigarettes Per Day | rs56116178 | FAM163B | G | 110.423 | 0.070 | 0.009 | -0.005 | 0.024 |
| Cigarettes Per Day | rs57708073 | ADAMTS7 | G | 156.547 | -0.051 | 0.006 | 0.022 | 0.015 |
| Cigarettes Per Day | rs58379124 | CHRNB3 | C | 153.804 | 0.067 | 0.007 | -0.020 | 0.016 |
| Cigarettes Per Day | rs6119248 | NOL4L | G | 177.972 | 0.033 | 0.006 | -0.015 | 0.014 |
| Cigarettes Per Day | rs632811 | RNF111 | G | 155.994 | -0.037 | 0.006 | 0.025 | 0.014 |
| Cigarettes Per Day | rs73229090 | GULOP | A | 114.119 | 0.055 | 0.009 | -0.013 | 0.022 |
| Cigarettes Per Day | rs7431710 | SLC25A20 | A | 172.110 | -0.035 | 0.006 | 0.011 | 0.014 |
| Cigarettes Per Day | rs75494138 | AMBRA1 | T | 94.623 | 0.060 | 0.011 | 0.017 | 0.030 |
| Cigarettes Per Day | rs790564 | RP11-32K4.1 | C | 161.471 | -0.041 | 0.006 | 0.042 | 0.015 |
| Cigarettes Per Day | rs7928017 | DRD2 | A | 179.904 | -0.033 | 0.006 | -0.005 | 0.014 |
| Cigarettes Per Day | rs79409323 | MORF4L1 | C | 60.394 | -0.119 | 0.017 | -0.022 | 0.048 |
| Cigarettes Per Day | rs7951365 | SOX6 | C | 167.565 | 0.039 | 0.006 | 0.008 | 0.014 |
| Cigarettes Per Day | rs8034191 | HYKK | C | 169.800 | 0.183 | 0.006 | 0.026 | 0.014 |
| Cigarettes Per Day | rs806789 | HIST1H3E | A | 180.744 | -0.031 | 0.006 | -0.010 | 0.013 |
| Smoking intiation | rs10001365 | TTC29 | A | 274.600 | -0.025 | 0.000 | -0.008 | 0.013 |
| Smoking intiation | rs1004787 | LINC01833 | A | 279.994 | 0.030 | 0.000 | 0.003 | 0.013 |
| Smoking intiation | rs10233018 | AC007568.1 | G | 281.095 | 0.027 | 0.000 | -0.029 | 0.013 |
| Smoking intiation | rs10279261 | EXOC4 | A | 273.019 | -0.021 | 0.000 | -0.012 | 0.014 |
| Smoking intiation | rs10490159 | AC007682.1 | T | 275.207 | 0.022 | 0.000 | -0.007 | 0.014 |
| Smoking intiation | rs10498846 | RNU7-66P | T | 281.237 | 0.021 | 0.000 | -0.014 | 0.013 |
| Smoking intiation | rs1050847 | ZCCHC14 | T | 278.626 | -0.022 | 0.000 | -0.013 | 0.014 |
| Smoking intiation | rs10905461 | RP11-575N15.1 | C | 241.244 | -0.024 | 0.000 | -0.003 | 0.015 |
| Smoking intiation | rs10945141 | ADGRB3 | A | 246.722 | 0.022 | 0.000 | 0.008 | 0.015 |
| Smoking intiation | rs11078713 | CHD3 | G | 277.337 | -0.020 | 0.000 | 0.016 | 0.014 |
| Smoking intiation | rs11135030 | RP11-524N5.1 | T | 258.490 | 0.023 | 0.000 | 0.019 | 0.014 |
| Smoking intiation | rs1154693 | RP11-384F7.1 | G | 203.564 | 0.033 | 0.000 | -0.004 | 0.019 |
| Smoking intiation | rs11658881 | SMG6 | G | 276.949 | 0.020 | 0.000 | -0.023 | 0.014 |
| Smoking intiation | rs11712680 | LINC02050 | C | 218.409 | -0.027 | 0.000 | 0.004 | 0.017 |
| Smoking intiation | rs117143374 | PSMG1 | C | 189.780 | 0.029 | 0.000 | 0.021 | 0.020 |
| Smoking intiation | rs11721059 | GRM7 | T | 280.626 | 0.020 | 0.000 | 0.004 | 0.013 |
| Smoking intiation | rs11768481 | DLX6-AS1 | A | 265.680 | -0.023 | 0.000 | 0.014 | 0.015 |
| Smoking intiation | rs117695734 | SDHD | G | 113.467 | -0.055 | 0.000 | 0.087 | 0.040 |
| Smoking intiation | rs11872397 | ZNF407 | A | 244.206 | -0.025 | 0.000 | -0.021 | 0.016 |
| Smoking intiation | rs11873164 | SETBP1 | T | 201.942 | -0.028 | 0.000 | -0.020 | 0.019 |
| Smoking intiation | rs12027999 | UBAP2L | C | 187.290 | -0.033 | 0.000 | -0.003 | 0.021 |
| Smoking intiation | rs12042107 | BARHL2 | C | 280.245 | -0.022 | 0.000 | -0.019 | 0.014 |
| Smoking intiation | rs12112638 | AUTS2 | G | 247.334 | -0.025 | 0.000 | 0.006 | 0.015 |
| Smoking intiation | rs12186738 | RP11-6N13.1 | T | 199.177 | -0.033 | 0.000 | 0.009 | 0.019 |
| Smoking intiation | rs12333760 | GS1-259H13.13 | C | 208.272 | -0.029 | 0.000 | 0.024 | 0.018 |
| Smoking intiation | rs12441907 | BNC1 | A | 221.104 | -0.029 | 0.000 | -0.017 | 0.017 |
| Smoking intiation | rs12474587 | SLC4A10 | T | 279.138 | 0.028 | 0.000 | 0.001 | 0.013 |
| Smoking intiation | rs12545053 | RP11-32K4.1 | G | 274.968 | 0.020 | 0.000 | 0.009 | 0.014 |
| Smoking intiation | rs12632110 | SEMA3F | G | 266.451 | -0.023 | 0.000 | -0.011 | 0.014 |
| Smoking intiation | rs12662631 | RP11-374I15.1 | T | 260.829 | 0.021 | 0.000 | 0.017 | 0.015 |
| Smoking intiation | rs12923427 | XYLT1 | T | 228.701 | -0.024 | 0.000 | -0.023 | 0.016 |
| Smoking intiation | rs13030994 | RPL6P5 | A | 281.183 | 0.036 | 0.000 | 0.015 | 0.013 |
| Smoking intiation | rs13136052 | RP11-98O2.1 | T | 278.778 | -0.020 | 0.000 | 0.007 | 0.014 |
| Smoking intiation | rs13261666 | TOX | T | 281.203 | -0.027 | 0.000 | -0.023 | 0.014 |
| Smoking intiation | rs134529 | TTC28 | C | 273.158 | -0.020 | 0.000 | -0.010 | 0.014 |
| Smoking intiation | rs1385108 | CTB-95D12.1 | T | 240.567 | 0.025 | 0.000 | -0.017 | 0.016 |
| Smoking intiation | rs1435741 | SEMA6D | A | 278.469 | 0.029 | 0.000 | 0.004 | 0.013 |
| Smoking intiation | rs1445649 | KCNJ3 | C | 280.509 | 0.024 | 0.000 | 0.013 | 0.013 |
| Smoking intiation | rs1518393 | VRK2 | C | 273.295 | 0.021 | 0.000 | -0.014 | 0.014 |
| Smoking intiation | rs17234745 | AKAP8P1 | C | 198.049 | -0.029 | 0.000 | -0.006 | 0.018 |
| Smoking intiation | rs17534089 | EFNA5 | G | 192.811 | -0.032 | 0.000 | -0.024 | 0.019 |
| Smoking intiation | rs1883526 | NA | C | 119.656 | 0.046 | 0.000 | 0.022 | 0.034 |
| Smoking intiation | rs1899896 | RP11-777J24.1 | T | 257.266 | 0.026 | 0.000 | -0.006 | 0.015 |
| Smoking intiation | rs2046850 | SYT14 | T | 223.286 | -0.025 | 0.000 | 0.004 | 0.017 |
| Smoking intiation | rs2276825 | TMEM110-MUSTN1 | C | 240.567 | 0.023 | 0.000 | -0.008 | 0.015 |
| Smoking intiation | rs2344508 | FPGT-TNNI3K | A | 279.069 | -0.022 | 0.000 | -0.004 | 0.013 |
| Smoking intiation | rs2378662 | RP11-158D2.2 | A | 280.382 | 0.021 | 0.000 | 0.004 | 0.014 |
| Smoking intiation | rs240963 | REV3L | C | 206.728 | -0.041 | 0.000 | -0.008 | 0.018 |
| Smoking intiation | rs266047 | AC092568.1 | A | 267.476 | -0.031 | 0.000 | 0.007 | 0.013 |
| Smoking intiation | rs27003 | MCTP1 | C | 260.829 | 0.024 | 0.000 | 0.041 | 0.015 |
| Smoking intiation | rs292071 | RP11-123O22.1 | C | 249.425 | 0.024 | 0.000 | 0.008 | 0.015 |
| Smoking intiation | rs3001723 | PTPRF | A | 256.514 | 0.034 | 0.000 | 0.012 | 0.015 |
| Smoking intiation | rs301805 | RERE | G | 276.748 | 0.021 | 0.000 | -0.021 | 0.014 |
| Smoking intiation | rs35702515 | THSD7B | T | 236.347 | 0.025 | 0.000 | -0.010 | 0.017 |
| Smoking intiation | rs359240 | AC007381.3 | A | 275.207 | 0.020 | 0.000 | -0.019 | 0.014 |
| Smoking intiation | rs3800227 | FOXO3 | G | 246.414 | 0.023 | 0.000 | 0.001 | 0.015 |
| Smoking intiation | rs3904512 | TRPC4 | A | 279.594 | -0.021 | 0.000 | -0.005 | 0.013 |
| Smoking intiation | rs4044321 | TENM2 | G | 269.492 | -0.028 | 0.000 | -0.012 | 0.014 |
| Smoking intiation | rs4236259 | ELFN1 | G | 281.158 | -0.025 | 0.000 | -0.002 | 0.014 |
| Smoking intiation | rs4523689 | OR10A6 | G | 274.475 | -0.021 | 0.000 | -0.007 | 0.014 |
| Smoking intiation | rs4543592 | CARM1P1 | C | 280.698 | 0.022 | 0.000 | -0.005 | 0.013 |
| Smoking intiation | rs4549681 | SDK1 | G | 267.555 | -0.024 | 0.000 | -0.006 | 0.013 |
| Smoking intiation | rs4571506 | CTC-498M16.4 | T | 280.197 | -0.028 | 0.000 | -0.012 | 0.013 |
| Smoking intiation | rs4674993 | NYAP2 | G | 225.412 | -0.025 | 0.000 | -0.021 | 0.017 |
| Smoking intiation | rs4785836 | LINC00922 | C | 273.295 | -0.020 | 0.000 | -0.010 | 0.014 |
| Smoking intiation | rs56820925 | RP13-379L11.3 | T | 257.914 | -0.022 | 0.000 | -0.002 | 0.014 |
| Smoking intiation | rs62098013 | DCC | A | 270.636 | 0.020 | 0.000 | 0.001 | 0.014 |
| Smoking intiation | rs6265 | BDNF | T | 218.409 | -0.032 | 0.000 | 0.014 | 0.017 |
| Smoking intiation | rs6433897 | LINC01934 | C | 246.414 | 0.022 | 0.000 | 0.018 | 0.015 |
| Smoking intiation | rs6669839 | ELAVL4 | T | 227.499 | 0.026 | 0.000 | -0.028 | 0.017 |
| Smoking intiation | rs6728726 | TMEM18 | C | 211.285 | 0.035 | 0.000 | 0.019 | 0.017 |
| Smoking intiation | rs6893752 | NDUFAF2 | G | 245.478 | -0.024 | 0.000 | 0.001 | 0.015 |
| Smoking intiation | rs6948707 | MAD1L1 | G | 277.616 | 0.022 | 0.000 | -0.007 | 0.013 |
| Smoking intiation | rs7024924 | PTPRD | C | 213.241 | 0.026 | 0.000 | 0.008 | 0.018 |
| Smoking intiation | rs7197072 | WDR90 | T | 239.882 | -0.025 | 0.000 | 0.001 | 0.016 |
| Smoking intiation | rs7224742 | C17orf75 | T | 273.566 | -0.021 | 0.000 | 0.005 | 0.014 |
| Smoking intiation | rs7322872 | CLYBL | T | 230.687 | -0.026 | 0.000 | -0.011 | 0.017 |
| Smoking intiation | rs7555507 | LINC01360 | T | 281.203 | -0.024 | 0.000 | 0.003 | 0.013 |
| Smoking intiation | rs76214862 | RP11-148E17.1 | C | 219.897 | -0.025 | 0.000 | -0.012 | 0.017 |
| Smoking intiation | rs7631735 | CADM2 | T | 274.362 | 0.020 | 0.000 | 0.011 | 0.014 |
| Smoking intiation | rs76608582 | CTB-50L17.10 | A | 121.068 | -0.050 | 0.000 | 0.029 | 0.038 |
| Smoking intiation | rs7901883 | BTRC | A | 235.251 | -0.025 | 0.000 | -0.023 | 0.016 |
| Smoking intiation | rs7929518 | EED | G | 233.384 | 0.024 | 0.000 | -0.002 | 0.016 |
| Smoking intiation | rs7938812 | NCAM1 | G | 274.968 | 0.044 | 0.000 | 0.005 | 0.013 |
| Smoking intiation | rs8039513 | SEMA6D | G | 220.661 | 0.026 | 0.000 | -0.025 | 0.017 |
| Smoking intiation | rs846799 | ASCC3 | G | 281.234 | -0.021 | 0.000 | 0.000 | 0.013 |
| Smoking intiation | rs9401770 | RP11-436D23.1 | A | 250.870 | 0.028 | 0.000 | 0.001 | 0.015 |
| Smoking intiation | rs993700 | AC112518.3 | C | 233.005 | -0.026 | 0.000 | 0.008 | 0.016 |
| Smoking Cessation | rs3025388 | DBH | G | 146.903 | -0.037 | 0.000 | 0.022 | 0.019 |
| Smoking Cessation | rs3866543 | ISL2 | G | 193.543 | 0.030 | 0.000 | -0.006 | 0.013 |
| Smoking Cessation | rs518425 | CHRNA5 | G | 175.288 | -0.037 | 0.000 | -0.022 | 0.015 |
| Smoking Cessation | rs56113850 | CYP2A6 | C | 192.301 | -0.055 | 0.000 | 0.004 | 0.015 |
| Smoking Cessation | rs6011779 | CHRNA4 | T | 150.309 | -0.055 | 0.000 | 0.000 | 0.017 |
| Smoking Cessation | rs7127006 | SOX6 | A | 172.630 | 0.032 | 0.000 | 0.005 | 0.015 |
| Smoking Cessation | rs9607805 | PHF5A | T | 172.630 | 0.034 | 0.000 | -0.017 | 0.015 |
| Alcohol consumption | rs10085696 | AUTS2 | G | 400.847 | -0.016 | 0.000 | -0.003 | 0.017 |
| Alcohol consumption | rs10753661 | LMX1A | A | 481.913 | -0.011 | 0.000 | -0.013 | 0.014 |
| Alcohol consumption | rs11860773 | RPSAP56 | C | 409.196 | -0.015 | 0.000 | 0.003 | 0.018 |
| Alcohol consumption | rs1229984 | ADH1B | C | 161.851 | 0.188 | 0.000 | -0.064 | 0.035 |
| Alcohol consumption | rs1260326 | GCKR | C | 503.946 | 0.024 | 0.000 | 0.008 | 0.014 |
| Alcohol consumption | rs1302808 | MANBA | A | 418.507 | 0.024 | 0.000 | 0.025 | 0.018 |
| Alcohol consumption | rs13107325 | SLC39A8 | T | 255.567 | -0.036 | 0.000 | 0.049 | 0.026 |
| Alcohol consumption | rs153106 | IL27 | C | 510.574 | -0.014 | 0.000 | 0.007 | 0.014 |
| Alcohol consumption | rs16854020 | BEND4 | A | 344.020 | 0.018 | 0.000 | 0.018 | 0.021 |
| Alcohol consumption | rs17177078 | TNRC6A | T | 244.987 | -0.025 | 0.000 | 0.011 | 0.027 |
| Alcohol consumption | rs17542254 | CLDN25 | G | 465.972 | 0.013 | 0.000 | -0.001 | 0.015 |
| Alcohol consumption | rs2299409 | ORC5 | A | 517.207 | -0.011 | 0.000 | -0.009 | 0.013 |
| Alcohol consumption | rs28680958 | ZBTB37 | A | 422.224 | -0.014 | 0.000 | 0.002 | 0.016 |
| Alcohol consumption | rs28712821 | KLB | A | 506.555 | 0.028 | 0.000 | 0.005 | 0.014 |
| Alcohol consumption | rs28732378 | CADM2 | G | 456.462 | -0.017 | 0.000 | -0.016 | 0.015 |
| Alcohol consumption | rs28929474 | SERPINA1 | T | 140.062 | -0.048 | 0.000 | -0.007 | 0.051 |
| Alcohol consumption | rs331939 | INPP4B | A | 492.802 | -0.012 | 0.000 | -0.001 | 0.014 |
| Alcohol consumption | rs34121753 | DNAH2 | G | 512.629 | 0.011 | 0.000 | -0.001 | 0.013 |
| Alcohol consumption | rs34704785 | LINC00364 | T | 516.758 | -0.011 | 0.000 | -0.013 | 0.013 |
| Alcohol consumption | rs3814877 | FAM57B | T | 507.165 | -0.011 | 0.000 | 0.002 | 0.014 |
| Alcohol consumption | rs4233567 | ARHGAP15 | T | 497.300 | -0.013 | 0.000 | 0.002 | 0.014 |
| Alcohol consumption | rs4309187 | DRD2 | C | 479.028 | 0.015 | 0.000 | 0.013 | 0.014 |
| Alcohol consumption | rs4752999 | SLC39A13 | T | 483.072 | -0.015 | 0.000 | -0.011 | 0.014 |
| Alcohol consumption | rs4916723 | LINC00461 | C | 511.991 | -0.011 | 0.000 | -0.003 | 0.014 |
| Alcohol consumption | rs528301 | LINC01833 | A | 513.085 | 0.016 | 0.000 | 0.005 | 0.013 |
| Alcohol consumption | rs55872084 | SGCD | T | 440.838 | 0.013 | 0.000 | 0.025 | 0.016 |
| Alcohol consumption | rs55932213 | LINC01505 | G | 451.262 | 0.012 | 0.000 | -0.008 | 0.015 |
| Alcohol consumption | rs55938136 | LINC02210-CRHR1 | G | 448.893 | -0.018 | 0.000 | 0.146 | 0.036 |
| Alcohol consumption | rs6106989 | ACSS1 | A | 504.195 | 0.011 | 0.000 | 0.011 | 0.014 |
| Alcohol consumption | rs6573197 | PSMA3 | T | 467.970 | -0.012 | 0.000 | 0.020 | 0.015 |
| Alcohol consumption | rs6739804 | EHBP1 | C | 480.301 | -0.013 | 0.000 | 0.000 | 0.014 |
| Alcohol consumption | rs682011 | SORL1 | C | 513.938 | 0.011 | 0.000 | -0.007 | 0.013 |
| Alcohol consumption | rs6951574 | DPP6 | C | 516.592 | 0.013 | 0.000 | -0.004 | 0.014 |
| Alcohol consumption | rs7106546 | snoU13 | T | 436.930 | 0.013 | 0.000 | -0.005 | 0.015 |
| Alcohol consumption | rs75120545 | AC019129.1 | T | 176.271 | -0.033 | 0.000 | 0.014 | 0.042 |
| Alcohol consumption | rs76640332 | KANSL1 | A | 418.507 | -0.021 | 0.000 | 0.116 | 0.016 |
| Alcohol consumption | rs838145 | IZUMO1 | A | 515.417 | -0.016 | 0.000 | -0.017 | 0.014 |
| Coffee consumption | rs10127720 | RP11-286B14.1 | T | 561.977 | -0.012 | 0.002 | -0.010 | 0.015 |
| Coffee consumption | rs1057868 | POR | C | 581.371 | -0.019 | 0.002 | 0.032 | 0.015 |
| Coffee consumption | rs10865548 | TMEM18 | A | 485.123 | -0.016 | 0.002 | -0.022 | 0.017 |
| Coffee consumption | rs10997940 | MYPN | C | 629.609 | 0.009 | 0.002 | 0.021 | 0.014 |
| Coffee consumption | rs11518562 | CD5 | C | 229.741 | -0.024 | 0.004 | 0.011 | 0.041 |
| Coffee consumption | rs117810762 | SCART1 | G | 168.381 | -0.034 | 0.006 | -0.027 | 0.051 |
| Coffee consumption | rs1260326 | GCKR | T | 628.544 | -0.013 | 0.002 | -0.008 | 0.014 |
| Coffee consumption | rs1545535 | RP11-507K12.1 | G | 597.889 | -0.012 | 0.002 | 0.006 | 0.014 |
| Coffee consumption | rs16903275 | LINC00461 | C | 467.079 | -0.016 | 0.002 | -0.008 | 0.018 |
| Coffee consumption | rs17687539 | STEAP2-AS1 | A | 521.706 | 0.010 | 0.002 | 0.002 | 0.017 |
| Coffee consumption | rs2231142 | ABCG2 | G | 408.421 | 0.015 | 0.002 | 0.013 | 0.021 |
| Coffee consumption | rs2330783 | SPECC1L | G | 150.616 | 0.044 | 0.007 | -0.046 | 0.054 |
| Coffee consumption | rs2465037 | RP11-228O6.2 | C | 607.977 | 0.011 | 0.002 | 0.010 | 0.014 |
| Coffee consumption | rs2472297 | CYP1A1 | C | 569.741 | -0.045 | 0.002 | -0.017 | 0.016 |
| Coffee consumption | rs2613458 | RP11-136I14.5 | A | 572.049 | -0.010 | 0.002 | -0.007 | 0.015 |
| Coffee consumption | rs2667773 | RP11-307C19.2 | A | 595.234 | 0.010 | 0.002 | 0.012 | 0.014 |
| Coffee consumption | rs34060476 | MLXIPL | A | 437.892 | -0.018 | 0.002 | -0.013 | 0.021 |
| Coffee consumption | rs34848782 | CLK3 | C | 235.974 | 0.025 | 0.004 | 0.034 | 0.041 |
| Coffee consumption | rs4410790 | AC003075.4 | T | 619.299 | -0.038 | 0.002 | 0.011 | 0.014 |
| Coffee consumption | rs56094641 | FTO | A | 630.518 | -0.015 | 0.002 | -0.020 | 0.013 |
| Coffee consumption | rs56113850 | CYP2A6 | T | 635.503 | -0.012 | 0.002 | -0.004 | 0.015 |
| Coffee consumption | rs574367 | SEC16B | G | 525.774 | -0.011 | 0.002 | -0.003 | 0.017 |
| Coffee consumption | rs57918684 | MED13 | G | 464.159 | -0.013 | 0.002 | -0.011 | 0.018 |
| Coffee consumption | rs6062357 | PCMTD2 | C | 630.880 | -0.010 | 0.002 | -0.003 | 0.013 |
| Coffee consumption | rs61182982 | CCDC33 | C | 463.322 | 0.014 | 0.002 | 0.001 | 0.018 |
| Coffee consumption | rs62106258 | AC105393.2 | T | 276.030 | 0.022 | 0.004 | 0.041 | 0.036 |
| Coffee consumption | rs660550 | SLC44A4 | C | 642.155 | 0.009 | 0.002 | -0.002 | 0.013 |
| Coffee consumption | rs66723169 | RP11-795H16.3 | C | 540.530 | -0.015 | 0.002 | 0.008 | 0.016 |
| Coffee consumption | rs72722581 | LINC01645 | T | 374.893 | -0.015 | 0.003 | -0.021 | 0.023 |
| Coffee consumption | rs73073176 | AC017060.1 | C | 429.760 | 0.023 | 0.002 | 0.032 | 0.020 |
| Coffee consumption | rs75347775 | GDF15 | G | 552.769 | -0.010 | 0.002 | -0.008 | 0.015 |
| Coffee consumption | rs993885 | LINC02142 | G | 617.364 | 0.010 | 0.002 | 0.005 | 0.014 |
